# Analysis on the synergistic variation law of pile refreezing process and bearing capacity based on high-strain dynamic test method

**DOI:** 10.1038/s41598-023-46372-w

**Published:** 2023-11-04

**Authors:** Dezhong Yu, Yang Cao, Qianqian Zhao

**Affiliations:** 1 School of Urban Construction, Zhejiang Shuren University, Hangzhou, 310015 Zhejiang China; 2 School of Water Conservancy and Civil Engineering, Northeast Agricultural University, Harbin, 150080 Heilongjiang China

**Keywords:** Environmental sciences, Engineering

## Abstract

The high-strain dynamic test method possesses the attributes of speed, efficiency, and environmentally-friendly, low-carbon characteristics. To study the reliability of the high-strain dynamic test method in detecting the bearing capacity of pile foundation in permafrost regions, 4 test piles, each with a length of 15 m and a diameter of 1.0 m, were poured based on the actual bridge construction project in the permafrost region of Daxing'an Mountains in China. Based on the temperature data between the piles and soil, the refreezing state of the pile foundation was comprehensively judged. Subsequently, the static method was employed to assess the friction resistance values and single pile bearing capacity of each soil layer on the pile side under different freezing states before and after the pile foundation refreezing. Building upon these findings, the restrictive parameters for soil elastic limit $q_{(i)}$, soil resistance $R_{U} (i)$ and other parameters in the pile-soil model of high-strain dynamic test method are clarified. The test and analysis results indicate that under the condition that the ground temperature of frozen soil is about − 1.9 °C, the pile-soil refreezing takes about 120 days, and the pile-soil refreezing is a slow process; In CAPWAPC calculation program, after the values of soil parameters were constrained by the results of static-load test, the calculated curve is in satisfactory agreement with the measured curve, and the parameters of the pile-soil model are reliable, which can be used for the rapid detection of the ultimate bearing capacity of piles in permafrost regions; In the process of pile-soil refreezing, the change of temperature field affects the freezing strength between pile and soil, the process of pile-soil refreezing is positively correlated with the increase of pile foundation bearing capacity.

## Introduction

With the evolution of our era and the escalating complexity of construction requirements, the natural surface or shallow soil of many construction sites is unable to meet these requirements. As a result, deep foundations can only be used to transfer the load of buildings to deeper soil layers^1^. As a typical representative of deep foundation, pile foundation stand out as a quintessential choice and find wide applications in multifarious complex foundation scenarios. In challenging geological areas, such as collapsible soil^2^, expansive soil^3^, frozen soil^4^, the stress relationship between pile and soil will change with the change of soil engineering properties. Frozen soil, in particular, represents an anisotropic, heterogeneous, multiphase complexity, which is composed of solid soil particles, liquid water, gas and ice. The properties of these four components in frozen soil and their proportional relationship and interaction will affect the engineering properties of frozen soil^5^.

The intricate material structure of permafrost is destined to its complex physical and mechanical properties. When constructing pile foundation in permafrost regions, due to the influence of foundation excavation, concrete pouring and hydration heat of cement concrete, the original freezing state of soil is destroyed, which leads to the thawing of frozen soil, the decrease of strength and bearing capacity. Over time, following construction, the pile and the surrounding soil gradually refreeze under the action of permafrost ground temperature and atmospheric temperature, and the soil strength and bearing capacity increase^6^. Throughout the refreezing phase, the pile and the surrounding soil are connected as a whole to bear the external load under the cementation of ice. Given the distinct engineering characteristics of frozen soil, it is of great scientific significance for the design and detection of pile foundation in permafrost regions to accurately grasp the variation law of bearing capacity before and after refreezing of pile foundation.

In the realm of assessing the bearing capacity of pile foundation in frozen soil, Domaschuk et al.^7^ carried out multi-stage and lateral pile foundation load creep tests in frozen sand, and studied the variation of instantaneous displacement and creep of pile top. Biggar et al.^8^ meanwhile, delved into the bearing characteristics of pile foundation in salty frozen soil area through model test, and discussed the influence of soil salt content, backfill material around pile, soil temperature and other factors on pile-soil freezing force and bearing characteristics of pile foundation. Taking into consideration the rheological properties of frozen soil, Wu et al.^9^ simulated the change of bearing capacity of single pile during the freezing process of pile foundation in frozen soil area by numerical simulation. Wang et al.^10^ conducted a model test on the bearing capacity of permafrost pile foundation by using the fast method, and obtained the relationship between the freezing force of the pile, the vertical bearing capacity of the pile and the temperature. Furthermore, Wu et al.^11^ scrutinized the influence of pile foundation thawing process on the bearing capacity of single pile in frozen soil area, compared and analyzed the difference between dynamic-load and static-load conditions, and gave the calculation process of pile body temperature during the thawing process. In light of the collective research findings, the bearing capacity of pile foundation is mainly measured by static-load method and high-strain test method^12^. The static- load method serves as a direct approach, the Q–S curve under static load is obtained by the actual bearing capacity of the test pile (such as the anchor pile method), and the actual bearing capacity of the pile is obtained directly, and the accuracy of the results is high. However, because the method requires 4 auxiliary piles, the scale and cost of the test are relatively large. At the same time, the construction of these auxiliary piles introduces additional heat into the frozen soil, further disrupting its frozen state. To safeguard the integrity of the permafrost, this detection method is not recommended in the permafrost areas^13^. Compared with the static-load method, the high-strain dynamic test method is simple, fast and does not require auxiliary piles. In recent years, the engineering community^14^ has shown an increasing interest in dynamic testing methods within permafrost areas. The dynamic testing methods include CASE method and CAPWAP method. The Case method and the measured curve fitting method of high-strain dynamic testing of pile foundation bearing capacity are described in China 's ' Building Foundation Pile Testing Technical Specification JGJ106-2014 '^15^.

The measured curve fitting method used in the high-strain dynamic test method applies a more complex pile-soil mechanical model, with the measured force, velocity, or the upgoing wave chosen as the boundary condition for fitting^16–18^. During the test, the PDS-PS detector collects the relevant data from the pile body when the heavy hammer hits the top surface of the pile foundation using embedded strain sensor and acceleration sensor on the pile foundation. Subsequently, the mechanical model of the pile-soil and its parameter values are then inversely calculated through numerical solution of the wave equation and a calculation curve is fitted. The calculation curve should be consistent with the measured curve when fitting is completed to obtain the vertical bearing capacity of the pile^19,20^. In the research and application of high-strain test, numerous scholars have concentrated their efforts on enhancing the calculation algorithm of the measured fitting method. Zhou et al.^21^ and Chen et al.^22^ improved the fitting analysis program by introducing new parameters, such as radiation damping, residual stress and pile tip crack in soil model, and the softening performance of pile end bearing layer, etc. Meanwhile, Xia et al.^23^ proposed a novel research idea in high-strain dynamic testing, and considered the hammer-pile-soil system as a unified entity. The piling process of a diesel hammer is simulated using the enhanced continuous model and the optimized discrete mass elastic model. Likins et al.^24^ conducted the dynamic test data of 119 driven piles and 23 cast-in-place piles, and pointed out that for the ultimate bearing capacity of driven piles and cast-in-place piles, the analysis results of CAPWAP method are in satisfactory agreement with the results of static-load test. Notably, the accuracy of driven piles is slightly higher than that of field cast-in-place piles, and the ratio of CAPWAP method to static-load test results reaches 0.98, and the covariance reaches 0.169. These findings suggest a conservative tendency in the CAPWAP results. Furthermore, Rausche et al.^25^ developed a more versatile dynamic test signal for pile-soil model analysis based on Smith model for complex soil conditions, and gave a series of ways to optimize soil resistance, including the correction of damping parameters, the use of radiation damping model and the analysis of residual stress, the measured curve fitting demonstrated strong efficacy.

The measured curve fitting method amalgamates the Case method and the Smith method, and leveraging the more precise CAPWAPC calculation program for analysis. The soil parameter values employed in the Smith model within the program significantly influence the test results. Due to the high hammering force, the static resistance of the soil around the pile will enter a plastic working stage that does not change with the change of the velocity of the pile when the high-strain dynamic test method is used in the permafrost areas. This leads to the emergence of a conspicuous vertical fracture surface in the soil near the surface of the pile. Consequently, when isolating the pile-soil combination within the fracture surface as the subject of study, the state of the combination can still be regarded as an elastic state, so that the wave theory of one-dimensional elastic rod can be used to continue the analysis and calculation. Consequently, a portion of the soil attached to the pile will be regarded as the attached mass and additional impedance of the pile, and the soil outside the fracture surface is regarded as the external resistance acting on the research object. Therefore, as long as the development law of the external soil resistance can be mastered, the pile foundation-frozen soil combination can still be solved based on the one-dimensional wave equation problem with resistance participation^26–28^. Combining these insights with the research findings from^29–34^, when processing the data of the fitting curve method, it is necessary to establish the relationship between the soil parameters of the Smith model and the static-load test index. By referencing to the field static-load test results, the constraints of soil elastic limit $q_{(i)}$ and soil resistance $R_{U} (i)$ are clarified to reduce the randomness of parameter selection in the analysis process of the measured curve fitting method. Typically, the soil resistance value $R_{U} (i)$ of each layer of the pile-soil model input in the non-frozen soil area is usually found in the specification. However, in the low-temperature conditions of diverse frozen soils within permafrost areas, there is no relevant data to refer to the selection range of the lateral friction resistance of different soil layers after freezing. In such cases, it becomes necessary to use the lateral friction resistance value of the soil layer measured by the static load test to correct the parameters.

In summary, the complexity of the frozen soil environment leads to distinct bearing characteristics for pile foundations in frozen soil and thawed soil thawed soil conditions. In order to further study the scientific problem of the control relationship between the mechanical parameters of frozen soil and the quality coefficient of the fitting curve, this study relies on the actual project in the permafrost region, and adjusts the soil parameters of the high-strain dynamic test method with the static-load data of the pile foundation. To fulfill the research objective, a temperature monitoring system was set up at the test pile to comprehensively judge the refreezing process of the pile foundation, accurately carry out the high-strain dynamic test before and after the refreezing of the pile foundation, and reveal the synergistic variation law of soil refreezing and pile foundation bearing capacity in the permafrost areas. Pile-soil refreezing is a slow process, the research results of this paper can quickly detect the friction resistance of pile side and resistance of pile end in different freezing states of pile-soil, and provide reference for the evaluation of pile foundation bearing capacity and subsequent construction under similar frozen soil conditions.

## Materials

The test piles were situated in the island permafrost region of Daxing'an Mountains in Heilongjiang Province, China. Their coordinates are at a latitude of 52°N with an average altitude of approximately 450 m; the surface water belongs to the Huma River system; the annual average temperature is − 2.4 °C, the average frost-free period is 98 days, the effective accumulated temperature of 10 °C ranging from 1276 to 1969 °C, the thickness of permafrost layer is 32 m, and the upper limit of permafrost is 2.1 m from the surface. The distribution of each soil layer on the pile side is shown in Table 1.

**Table 1:** The distribution of soil.

Soil layer number	Name	Thickness (m)	Water content (%)
1	Filled soil	2.1	5.0
2	Peat soil	0.5	13.4
3	Silty clay	1	18.3
4	Rounded gravel	1.3	13.7
5	Round gravel mixed with soil	2	14.8
6	Stone clip soil	4	15.1
7	Strongly weathered tuff	2.7	6.5
8	Medium weathered tuff	1.4	6.5

To validate the applicability and reliability of the high-strain dynamic test method in permafrost areas, four test piles with a length of 15 m, a diameter of 1.0 m and a pile strength grade of C30 were poured in permafrost regions with the same geological conditions. The static-load test of the pile foundation before and after refreezing were carried out on test pile-1 and test pile-2 respectively; the high-strain dynamic test of the pile foundation before and after refreezing were carried out on test pile-3 and test pile-4 respectively. These four test piles were constructed in parallel, and the date of pile formations were the same. Therefore, only one of the test piles was equipped with a temperature monitoring system to monitor the temperature change of the pile foundation. Utilizing the temperature monitoring data, the refreezing process of the pile foundation is determined, and the time nodes of the static-load test and high-strain dynamic test before and after the refreezing of the pile foundation are determined.

## Test principle and method

### Test principle of high-strain dynamic test method (measured curve fitting method)

The curve fitting method uses numerical calculation of wave problem to calculate the pile-soil mechanical model and its parameters^35^. The specific analysis processes are as follows^36^:

#### Hypothesis of pile

The wave equation fitting method subdivides the pile into N_p_ member unit, each approximately 1 m in length. The assumptions include:

① The pile body is a continuous one-dimensional elastic rod.
② Compared with the piles, the unit area and elastic modulus are different.
③ Impedance changes at the cross-section of adjacent units.
④ Element lengths may vary, but the time Δt of stress wave passing through each unit must be equal.
⑤ Soil resistance exclusively exerts its influence at the base of each unit.

#### Fluctuation analysis

The fluctuation analysis involves pile model, soil resistance model and pile-soil interaction. The measured curve fitting method employs the continuous bar model described in CAPWAPC^37^.

The wave solution of the one-dimensional wave equation as shown in formula (1):

1 $$ f(x - ct) $$

The solution consisted of two parts, which represent two traveling waves respectively. Where, $f(x - ct)$ is the downward disturbance wave transmitted along the x-axis with the wave velocity $c$ along the rod axis direction; $g(x + ct)$ is the upward disturbance wave transmitted along the x-axis with the wave velocity $c$ along the rod axis direction.

The velocity of pile body particle as shown in formula (2):

2 $$ V(i,j) = \frac{{P_{d} (i,j)}}{{Z_{i} }} + \frac{{P_{u} (i,j)}}{{Z_{i + 1} }} $$

The displacement value of the pile body particle as shown in formula (3):

3 $$ S(i,j) = S(i,j - 1) + \frac{\Delta t}{2}[V(i,j - 1) + V(i,j)] $$

where $P_{u}$ is upward wave, $P_{d}$ is downward wave, $V$ is measured velocity wave, *Z* is wave impedance.

#### Pile-soil model

① Pile side soil static resistance model. The static resistance model of pile side soil involved in the measured curve fitting method adopts the soil resistance model of Smith method, as shown in formula (4):
  4 $$ Rs(i,j) = \frac{{R_{U} (i)}}{{q_{(i)} }}[S(i,j) - DE(i,j)] $$
  where $R_{U} (i)$ is the ultimate side resistance of the soil unit, $q_{(i)}$ is the pile side elastic limit of the soil unit, $S(i,j)$ is the maximum displacement of the soil unit, and $DE(i,j)$ is the plastic displacement of the soil unit.
② Pile side soil dynamic resistance model. In the high-strain detection, the pile is subjected to a dynamic impact load, and the soil loading process inevitably produces a certain dynamic effect. In order to describe the dynamic effect of soil, the Smith damping model is used, as shown in formula (5):
  5 $$ R_{d} (i,j) = \left| {\left. {R_{s} (i,j)} \right|} \right. \cdot J_{s} (i) \cdot V(i,j) $$
  where $J_{s} (i)$ is the Smith damping coefficient, $R_{s}$ is the static friction resistance of the pile side unit, $R_{d}$ is the dynamic friction resistance of the pile side unit.
③ Static resistance model of pile end soil. The traditional Smith static resistance model is used in the pile tip static resistance model. The difference between the pile end soil static resistance model and the pile side static resistance model is that the pile end static resistance takes into account the influence of the pile tip soil gap; the force between the pile end and the soil is formed by compression, so the pile end soil does not provide tension.
④ Pile end soil dynamic resistance model. The dynamic resistance model of pile tip soil adopts Smith dynamic resistance model. The calculation method is consistent with formula (5).

#### Curve fitting

In the fitting process, the main time period is divided into 4 sections:

① The length of time interval is 2L/c from the shock wave peak. During this period, the pile top mainly receives the stress echo generated by the pile side resistance. Consequently, the waveform of this time period is mainly used to correct the distribution of the pile side resistance.
② Commencing from the conclusion of the first time interval, the length of the time interval is t_r_ + 3 ms, where t_r_ is the time from the beginning of the shock wave to the peak of the velocity wave. During this period, the pile top began to receive the stress echo generated by the pile end. Therefore, this time period is mainly used to correct the value of pile end bearing capacity.
③ Following the end of the first time interval, the length of the time interval is t_r_ + 5 ms. In this period, the damping coefficient has a great influence on the waveform. Therefore, this time interval is mainly used to correct the damping coefficient value.
④ Commencing from the conclusion of the second time interval, the length of the time interval is 20 ms. During this period, the pile top begins to receive the stress echo generated by the unloading soil, so the waveform of this time section is mainly determined by the unloading stage of the soil, which is used to correct the parameters related to the unloading properties of the soil.

The calculation is started from the rising section of the curve. The calculation process is as follows: The force curve F_m_ (t) is derived from the velocity curve V_m_ (t), and the pile element number is represented by i, and the calculation time is represented by j.

① Division of pile element grid. The pile body is defined and subdivided into n sub-unit objects subordinate to the pile body. At the same time, the soil object should be specified for each pile unit.
② Initial assignment of pile-soil model parameters. At the initial moment, based on the geological data, engineering experience and in-situ test results, the initial values of $R_{U} (i)$, $q_{(i)}$, $J_{s} (i)$ and other parameters of each soil layer object are assigned. In the fitting process: $q_{(i)}$ generally takes a value between 0.025 mm and the maximum displacement of the element, and the initial value is 0.25 mm; $J_{s} (i)$ is 0.08–1.0 s/m, and the adjustment range is 0.02 s/m; $R_{U} (i)$ is assigned with reference to the static load test results, and the adjustment range of each soil layer parameter does not exceed ± 10% of the measured value^38^.
③ Based on the pile-soil model and iterative formula (6), the calculated Fc (t) is compared repeatedly with the measured value Fm(t). And the bearing capacity of pile foundation is determined by combining the error of the two and the quality of curve fitting. If the fitting curve is satisfactory, the fitting process concludes, and the model parameters of the fitting curve are assessed. If the model parameters are reasonable, the bearing capacity results can be output, otherwise the fitting still needs to be restarted. If the fitting effect is not ideal, then return step ②, adjust the parameters Based on the curve error, and restart the calculation until the fitting effect is satisfactory, and the Mq is required to be less than < 50^15^.
  6 $$ F(x) = \sum\limits_{i = 1}^{{N_{pile} }} {ABS[F{}_{c}(j) - F{}_{m}(j)]} $$
  where $$x = (R_{u} (i),Q(i),H_{s} (i),R_{t} ,Q_{t} ,f_{f} ,W_{s} ,J_{ms} )$$.

**Figure 1:**
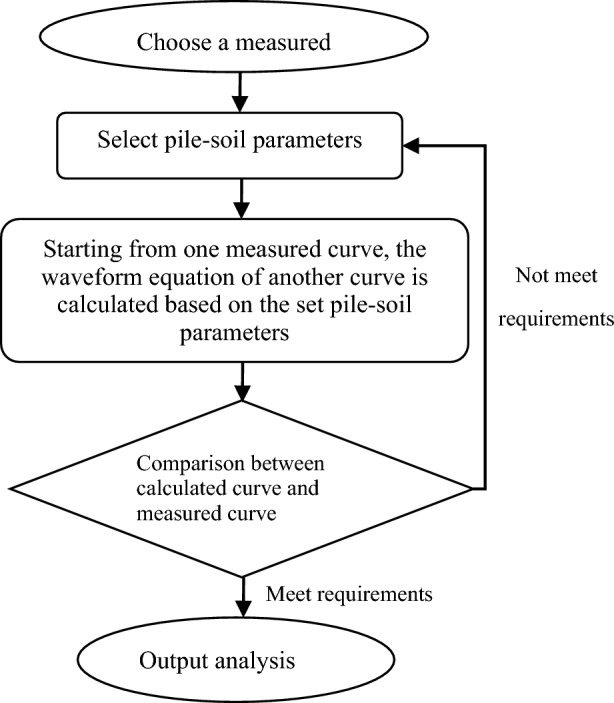
The calculation process of fitting method.

The fitting calculation process of CAPWAPC analysis program is shown in Fig. 1.

### Freezing characteristics of pile-soil interface

In the permafrost areas, with the decrease of temperature, the soil will freeze when it reaches its freezing point, leading to a gradual increase in freezing depth. During this process, the water in the soil will continue to migrate to the freezing front. Under vertical loading conditions, the bearing capacity of pile foundation in permafrost is mainly composed of two parts: the freezing force of soil around the pile and the resistance of frozen soil layer at the bottom of the pile^39^. The size of the freezing force is affected by the freezing strength. The freezing strength is not only related to the temperature, soil, water content, etc., but also closely related to the roughness of the structural contact surface. Freezing strength rises with an increase in the surface roughness. The freezing strength can be measured by the shear strength of the material contact interface^40,41^. Due to the uncertainty of soil freezing strength and pile surface roughness, the failure surface between pile and soil under high-strain vertical load becomes very complex. The accuracy of the fitting calculation of CAPWAPC analysis program is directly related to the strength parameters ( side friction resistance ) of each layer of soil. Therefore, in order to ensure the accuracy of the test results, the side friction resistance of the soil before and after freezing should be measured respectively, which provides a reference for the parameter setting of the soil in the pile-soil calculation model.

### Test equipment

**Figure 2:**
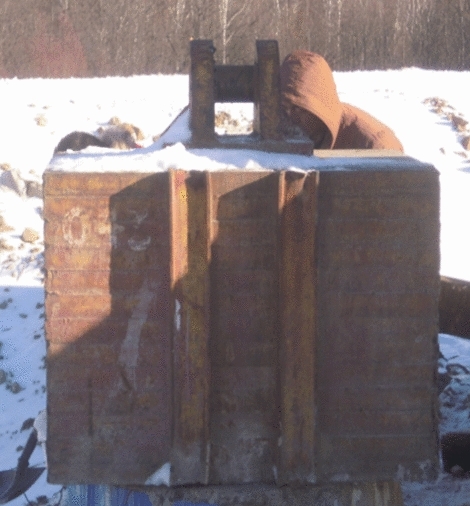
Vibration equipment.

**Figure 3:**
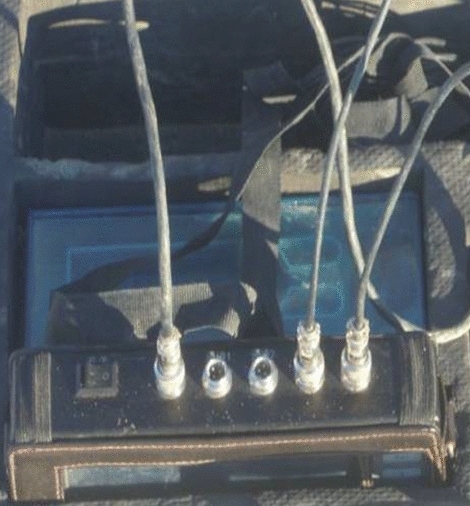
Data collecting instrument.

**Figure 4:**
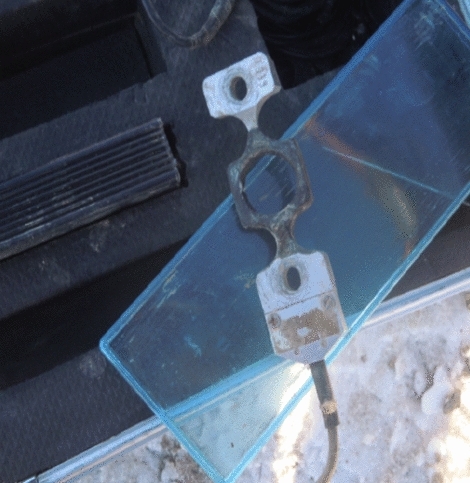
Strain sensor.

**Figure 5:**
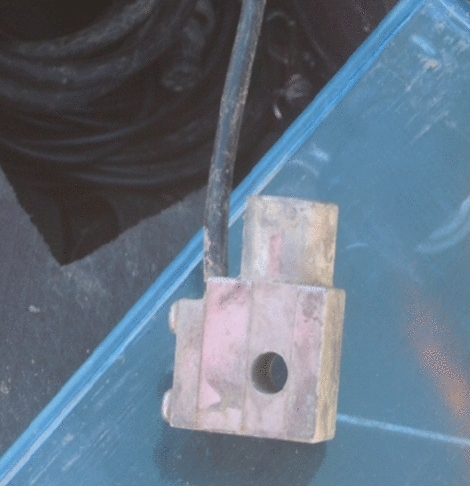
Acceleration sensor.

**Figure 6:**
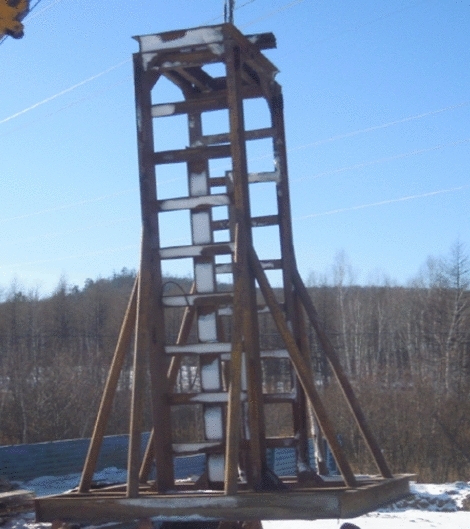
Guide frame.

**Figure 7:**
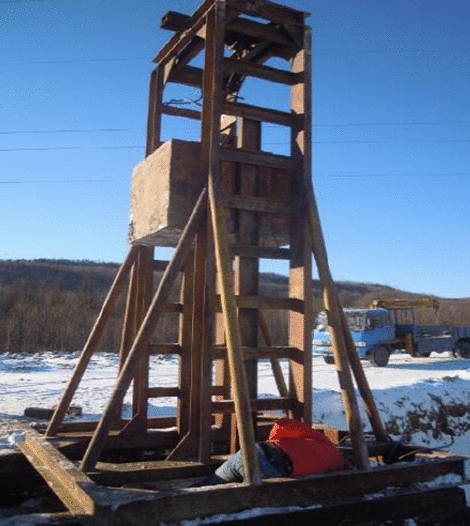
Layout diagram of dynamic equipment.

The equipment employed in the high-strain dynamic test method comprises an excitation equipment, guide frame, high-strain data acquisition instrument, acceleration sensor and strain sensor. The excitation equipment is a 5t hammer; The guide frame is a special iron frame with guide sliding to 4.5 m in height, which is used to guide the heavy hammer to fall freely. The high-strain data acquisition instrument employed in this method is a PDS-PS type detector, which is used to gather relevant data throughout the test. The acceleration sensor used is a SV-5 type, and the sensitivity is 5.8 mV/g. The strain sensor is a CYB-YB-F1K type. The field layout of the equipment is shown in Figs. 2, 3, 4, 5, 6, 7.

### Test method

Based on the data presented in Table 2, test pile-1 is employed for static load testing before pile foundation refreezing, and test pile-2 is used for static load testing after pile foundation refreezing. Based on geological survey data, the pile body comprises eight distinct soil (rock) layers. To assess the side friction resistance of different soil layers after freezing, two steel bar meters are symmetrically arranged at the interface of each layer of soil, so a total of 14 steel bar meters are arranged at seven interfaces of eight soil layers of a pile. For measuring the pile tip resistance, two pressure boxes are symmetrically arranged at the bottom of the pile. The load boxes are arranged at 2.5 m from the pile bottom. Test pile-3 is designated for dynamic testing before pile foundation refreezing, and test pile-4 is used for dynamic testing after pile foundation refreezing. Prior to testing, a pair of acceleration sensors and a pair of strain sensors should be arranged on the surface of the pile at a distance of 1.5 m from the top.

**Table 2:** Friction resistance of each soil layer.

Soil layer number	Name	The measured frictional resistance before and after refreezing (kPa)	
		Pile 1	Pile 2
1	Filled soil	24	37
2	Peat soil	22	28
3	Silty clay	31	38
4	Rounded gravel	90	130
5	Round gravel mixed with soil	70	115
6	Stone clip soil	73	125
7	Strongly weathered tuff	125	164
8	Medium weathered tuff	130	170

When the strength of the pile body concrete satisfies the testing requirements, the temperature monitoring system records the temperature of the pile foundation to guide the timing of the static and dynamic tests. As there is no existing data on the measured bearing capacity of pile foundation after refreezing in the Daxing'an Mountains area, the static-load test load is estimated to be 10,000kN, which is incrementally applied over 15 stages until the pile foundation reaches failure. Subsequently, the bearing capacity, pile side friction and pile end resistance of the refrozen pile foundation are then obtained. Based on the soil friction resistance measured by the static-load test, the setting parameters of the high-strain dynamic pile-soil mechanics model soil layer are corrected, a fitting curve is plotted, and the bearing capacity of the pile foundation is determined.

In contrast to regular soil, the mechanical properties of frozen soil under impact load show obvious strain rate effect, that is, the strength limit of frozen soil increases with the increase of loading strain rate, which is common in ice structure^42,43^. When the frozen soil is subjected to high-strain rate loads such as impact and explosion, the adiabatic temperature rise will occur inside the permafrost. The temperature field generated by the temperature rise causes the phase transition between ice and water and the change of ice content, thus affecting the mechanical properties of permafrost^44,45^. Zhang et al.^46^ proposed the strain rate- temperature equivalence of permafrost by regression analysis of test data, and used the damage caused by adiabatic temperature rise to describe the thermal softening effect of permafrost under impact load, and proposed the damage evolution model of permafrost. In order to minimize the influence of the temperature field generated by the temperature rise on the test results of the bearing capacity of the pile foundation, the test pile head should be reinforced and the pre-strike of the excitation equipment should be carried out before the formal high-strain test. Adjustments should be made to the hammer's drop height and various parameters within the data acquisition program. Following a waiting period, the bearing capacity of the high-strain pile foundation should be tested after judging the stability of the pile-soil temperature field based on the temperature monitoring data.

### Layout of pile temperature monitoring system

**Figure 8:**
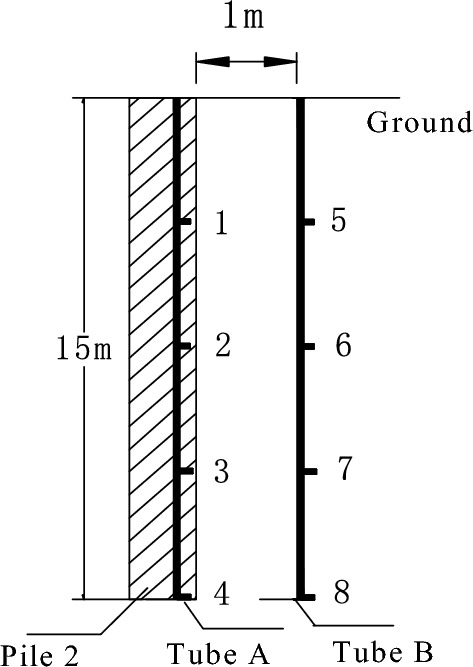
The facade of temperature observation system.

The temperature measuring tubes are installed inside the test pile-2, each placed 1 m from the edge of the pile foundation. Temperature measuring tube-A, with a length matching the pile length (L) at 15 m, was positioned 7 cm from the edge of the pile foundation. It is composed of four temperature sensors (NO. 1–4) in parallel, which are arranged at 1/4L (3.75 m), 1/2L (7.5 m), 3/4L (11.25 m) and the bottom of the pile (15 m) from the top of the pile. The temperature measuring tube-B is installed at a distance of 1 m from the edge of the pile foundation. The length and temperature sensor (NO.5–8) are arranged at the same position as the temperature measuring tube A. The elevation layout is as shown in Fig. 8.

## Results

### Temperature monitoring results

**Figure 9:**
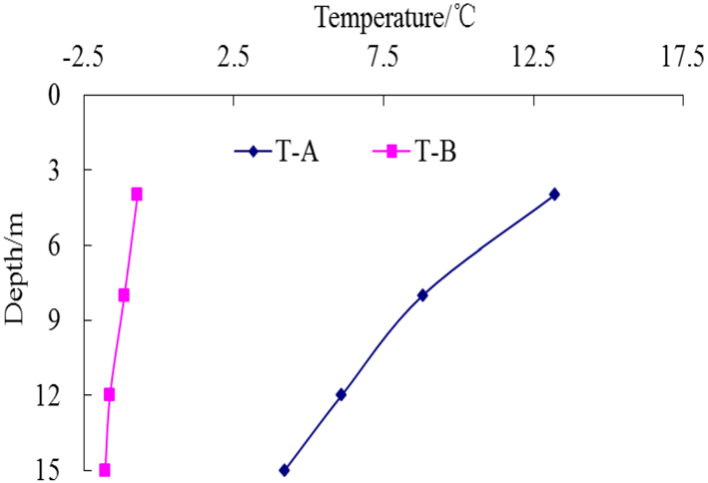
Temperature curve of pile after10 days.

**Figure 10:**
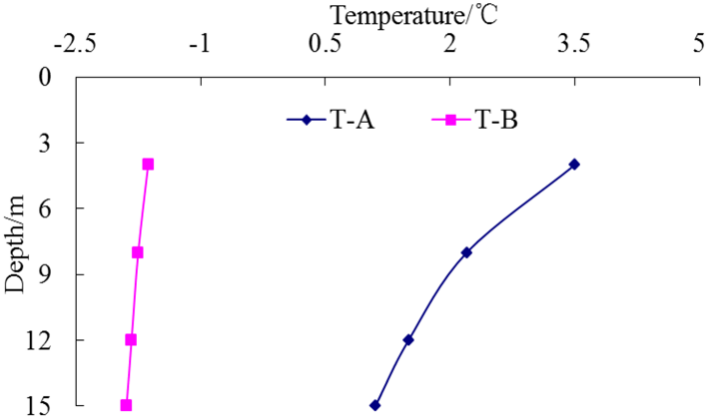
Temperature curve of pile after 30 days.

**Figure 11:**
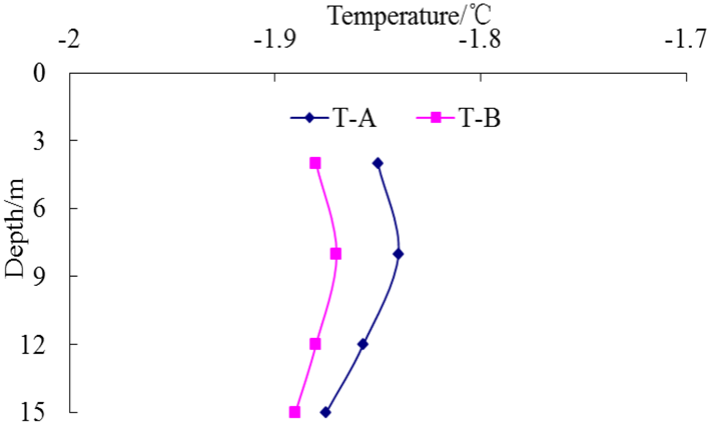
Temperature curve of pile after120 days.

**Figure 12:**
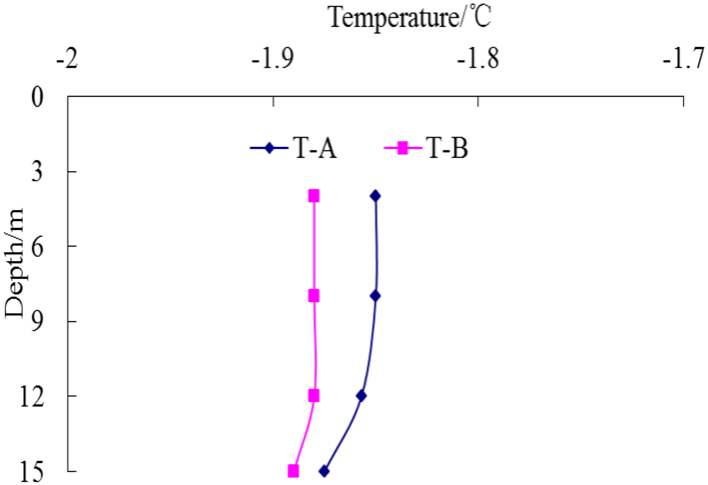
Temperature curve of pile after 150 days.

In the process of pile-soil refreezing, temperature data for each measuring point of temperature measuring tubes A and B are shown in Figs. 9, 10, 11, 12 respectively.

Temperature data recording by the monitoring system begins on the day of pile formation. As depicted in Fig. 10, the temperature of each measuring point of the pile foundation remains above 0 °C, indicating that the pile foundation has not yet begun to freeze. Concurrently, the compressive strength of the concrete test block is 26.4 MPa, meeting the test requirements and exceeding 80% of the design strength of the pile body concrete. The static load test of the test pile-1 before the frozen soil refreezing and the high-strain dynamic test of the test pile-3 before the frozen soil refreezing are carried out at this time.

Wu et al.^47^, Tang et al.^48^, Li et al.^49^, and Wang et al.^50^ conducted studies on the refreezing duration of pile foundation. The findings indicate that the refreezing time of pile foundation is affected by many factors, including pile foundation size, concrete molding temperature, and ground temperature of the frozen soil. Typically, the refreezing time of pile foundation is usually more than 100 days. Song et al.^51^ and Ketil et al.^52^ investigated the rate of ground temperature change in frozen soil. The findings reveal that the change rate of temperature difference between pile and soil is slow after the pile foundation in permafrost region is refrozen, and the change rate is usually 0.1–0.2 °C per decade. In accordance with relevant research findings, when the temperature difference between different temperature measuring points at the same depth of the temperature measuring tube A and the temperature measuring tube B is less than 0.1 °C, the soil is judged to complete the refreezing. Figures 11 and 12 illustrate that over time, the pile foundation gradually refroze under the action of permafrost ground temperature. Following 120 days, the internal temperature of the test pile body was basically consistent with the soil temperature at 1 m on the pile side. The temperature difference at the same depth was less than 0.1 °C, which was basically unchanged compared with the temperature after 150 days. Therefore, it was considered that the pile foundation completed the refreezing at 120 days, and the compressive strength of the concrete test block was 32.4 MPa, meeting the test criteria. Subsequently, the static-load test of test pile 2 after frozen soil refreezing and the dynamic-load test of test pile 4 after frozen soil refreezing were carried out.

### Bearing capacity test results

The bearing capacity of test pile-1 and test pile-2 are tested by self-balanced static-load test^53^, the self-balanced static-load test is to arrange the load-box at the calculated position inside the pile body (the load-box divides the pile into the upper pile and the lower pile); six electronic displacement meters are arranged on the top of the pile, of which two are used to measure the upward displacement of the load-box, two are used to measure the downward displacement of the load -box, and the remaining two are used to measure the upward displacement of the top surface of the pile foundation; two steel bar meters are symmetrically arranged at the interface of different soil layers to calculate the axial force of each pile body layer and the friction resistance of each soil layer on the pile side, according to the distribution of soil layer in Table 1, fourteen steel bar meters should be arranged in interfaces of 8 soil (rock) layers; two pressure-boxes are symmetrically arranged on the bottom surface of the pile foundation to calculate the pile tip resistance. When the static-load test was carried out, the load-box was filled with oil through the ground high-pressure oil pump, and the load-box transmited the force to the pile body. By loading to the limit state, the ultimate friction resistance of the pile side, the ultimate resistance of the pile end and the vertical ultimate bearing capacity of the pile can be detected.

During data processing, according to the axial force difference between the upper and lower interfaces of each layer of soil and formula 7, the pile side friction of each soil layer after pile foundation refreezing is calculated, the calculation results of pile side friction are shown in Table 2. According to the segmented load value obtained from the static-load test, the bearing capacity of the pile foundation is converted, the specific calculation method as shown in formula 8, the calculation results of bearing capacity are shown in Table 4.

7 $$ q_{s} $$

where $q_{s}$ is the friction resistance of each soil layer on the pile side (kN/m^2^); $\Delta Q_{z}$ is the difference of axial force between the measured sections of the pile (kN); $\Delta F$ is the lateral surface area of the pile section between the measured sections of the pile body (m^2^).

8 $$ P_{u} = \frac{{Q_{uu} - W}}{\gamma } + Q_{lu} $$

where $P_{u}$ is the vertical compressive ultimate bearing capacity of pile foundation (kN); ${\text{Q}}_{{{\text{uu}}}}$ is the ultimate load value of the upper pile (kN); ${\text{Q}}_{{{\text{lu}}}}$ is the ultimate load value of the lower pile (kN); ${\text{W}}$ is the self-weight of the upper pile (kN); $\gamma$ is the pile side friction correction coefficient of the upper pile, the value is 0.9.

The decrease of the temperature significantly augments the freezing strength of the pile-soil interface. Lower temperatures facilitate the complete freezing and crystallization of water within the soil, resulting in enhanced freezing at the pile-soil interface. Simultaneously, the horizontal frost heaving force generated in the soil is greater, which makes the pile-soil interface squeeze each other. The force is greater, prompting the freezing strength to increase. More fine ice particles are produced after the failure of the pile-soil interface, which increases the friction force between the pile-soil interface. Due to the existence of free water, the water will move closer to the pile-soil contact surface during the freezing process, leading to the formation of additional ice crystals at the interface, thereby reinforcing the bond between the pile and the soil, ultimately increasing the freezing strength and frictional resistance between the contact surfaces. From the data in Table 3, it can be seen that the temperature of pile-soil decreases continuously under the action of permafrost ground temperature, and the friction resistance of each soil layer increases. The law of synergistic change between temperature and friction resistance is presented.

**Table 3:** The pile penetration of dynamic-load test.

Pile number	Penetration before refreezing (mm)	Penetration after refreezing (mm)
3	3.21	–
4	–	2.84

**Figure 13:**
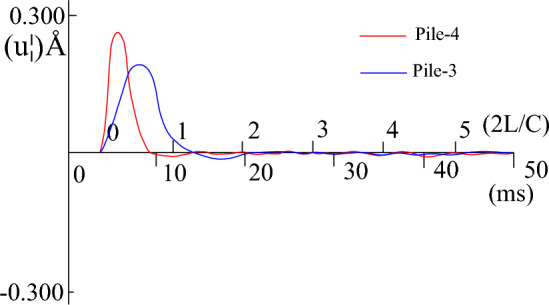
The curves of strain.

**Figure 14:**
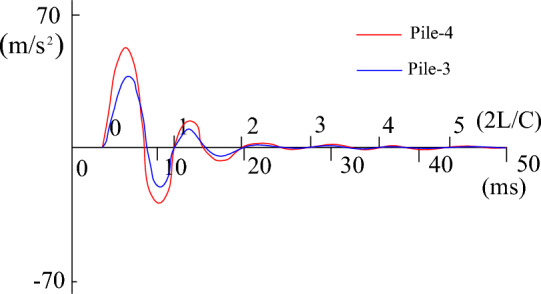
The curves of accelerating.

**Figure 15:**
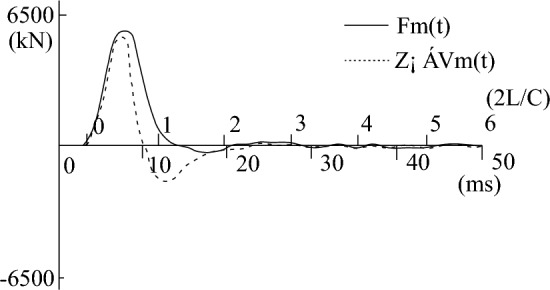
The curve of the measured force and measured speed of test pile-3 before frozen.

**Figure 16:**
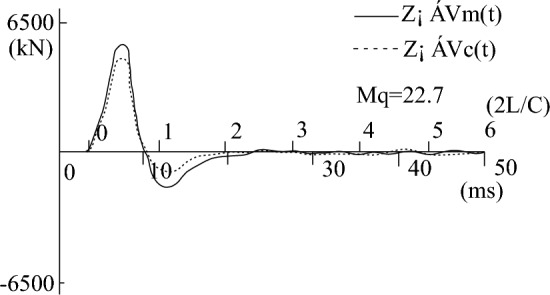
The curve of the calculated speed and measured speed of test pile-3 before frozen.

**Figure 17:**
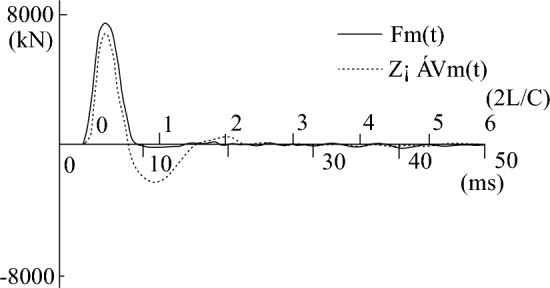
The curve of the measured force and measured speed of test pile-4 after frozen.

**Figure 18:**
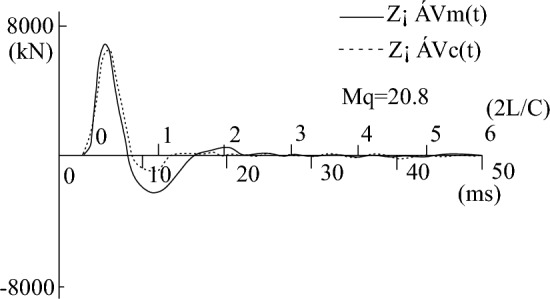
The curve of the calculated speed and measured speed of test pile-4 after frozen.

During the process of fitting the high-strain calculation curve to the measured curve, the relevant parameters of the pile-soil model soil layer in the CAPWAPC program are repeatedly adjusted (the adjustment range of each soil layer does not exceed 10% of the measured value) based on the lateral friction resistance of each soil layer measured by the static load test (as shown in Table 2). The penetration data collected by the dynamic test as shown in Table 3. The strain and acceleration test results are shown in Figs. 13 and 14, respectively. Based on the data presented in Figs. 13 and 14, formulas 9 and 10 are used to calculate $F_{{\text{m}}} {(}t{)}$ and $Z \cdot V\left( t \right)$ respectively. The measured force and the measured velocity curve, as well as the curve fitted by the calculation program are shown in Figs. 13, 14, 15, 16, 17, 18

9 $$ F_{m} (t) = \rho AC^{2} \varepsilon (t) $$

where $\rho$ is the pile density, kg/m^3^; $A$ is the cross-sectional area of the pile, (m^2^); $C$ is the propagation velocity of longitudinal wave in the pile (m/s).

10 $$ Z \cdot V\left( t \right) = \rho \cdot A \cdot c\int_{0}^{T} {a(t)dt} $$

wherewhere $$x = (R_{u} (i),Q(i),H_{s} (i),R_{t} ,Q_{t} ,f_{f} ,W_{s} ,J_{ms} )$$.

$Z$ is the wave impedance of pile body, (N s/m).

Figures 15, 16, 17, 18 depict the normal measured curve signals for each height strain measurement analysis of the test piles, and there is no curve distortion. The measured force curve and the velocity curve take-off point coincide and reach the peak at almost the same time, suggesting the normal operation of the sensor hammering system and the upper section of the pile top. Subsequently, due to the effect of soil friction resistance on the pile side, the measured force curve and the velocity curve are gradually separated. The fitted mass number Mq in Figs. 16 and 18 is less than 2.5 mm, which indicates that the soil resistance is fully excited. The comparison of static-load and dynamic-test results is shown in Table 4.

**Table 4:** The statistics of dynamic and static load test results.

Item	Before refreezing		After refreezing	
	Static load	High-strain dynamic	Static load	High-strain dynamic
Ultimate bearing capacity (kN)	5020	5237	7334	7183
Friction resistance of pile (kN)	3645	3628	5368	5383
Proportion of pile side friction resistance (%)	72.6	69.3	73.2	74.9
Dynamic and static bearing capacity ratio (%)	1.04		0.98	
Ultimate bearing capacity (%)	− 4.32		2.06	
Average error of ultimate bearing capacity (%)	3.19%			

From Table 4, it can be seen that the error of the ultimate bearing capacity measured by the measured curve fitting method and the self-balanced static-load method is less than 10% before and after the refreezing of the permafrost pile foundation, and the average error is 3.19%. On the basis of accurately grasping the value of the lateral friction resistance of each soil layer after freezing and correcting the relevant parameters of the soil layer in the pile-soil mechanics model, the ultimate bearing capacity of the pile foundation measured by the high-strain dynamic test method is consistent with the ultimate bearing capacity measured by the static load method.

From Figs. 9, 10, 11, 12, it can be seen that the temperature of the soil during the pile-soil refreezing process generally decreases with the increase of the buried depth of the pile, and the closer to the surface, the higher the temperature. Furthermore, there is a decreasing trend in temperature extending from the pile-soil contact point towards the periphery. The highest temperature is observed at the contact surface, while the temperature decreases as one moves farther away from this surface. The change law of temperature is consistent with the transfer law of external cooling capacity to soil, so a temperature gradient from outside to inside and from bottom to top is generated in the soil. The influence of temperature field on the strength of frozen soil is very important. Because the frozen soil itself is a combination of soil particles and ice crystals in the soil, the change of temperature field will inevitably cause the phase change of ice crystals in the soil, thus affecting the degree of cementation and structural relationship between the two, and changing the bearing capacity of the pile foundation. Based on the field high-strain dynamic test, the bearing capacity of the pile foundation is 5237 kN and the penetration is 3.12 mm before the pile-soil refreezing. After the pile-soil refreezing, the bearing capacity of the pile foundation is 7183 kN, and the penetration is 2.84 mm, indicating that the freezing force between the pile and the soil reduces the plastic settlement of the pile foundation and improves the bearing capacity of the pile foundation.

## Conclusions

Based on temperature tracking and monitoring of the test pile, the self-balanced static load and high-strain dynamic test of the bearing capacity of the pile foundation before and after refreezing are carried out. The following conclusions are drawn from a comparative analysis of the test results:

1. The mechanical properties of pile-soil in permafrost are closely related to the particle composition and freezing state of frozen soil. The interaction between pile-soil is affected by multi-field coupling such as force field and temperature field. Based on temperature monitoring results, it was observed that under the condition of frozen soil ground temperature of approximately − 1.9 °C, the refreezing of pile foundation takes about 120 days. After refreezing, the friction resistance ( freezing strength ) of each layer of soil increases to varying degrees, and the increase range is between 6 and 52 kPa.
2. When employing the high-strain dynamic test method (curve fitting method) for assessing the bearing capacity of pile foundation in frozen soil area, it is crucial to establish the relationship between pile-soil parameters and static load test indexes. Based on the results of field static load test, the parameter constraints such as soil elastic limit $q_{(i)}$ and soil resistance $R_{U} (i)$ are defined. After adjusting the values of each parameter in the CAPWAPC calculation program, the curve fitting mass Mq < 50, indicating that the calculated curve is in satisfactory agreement with the measured curve, and the parameters of the pile-soil model are reliable. This reliability makes them suitable for evaluating the ultimate bearing capacity of foundation piles in permafrost regions.
3. Variations in the temperature field induce phase changes in water–ice within the soil, consequently influencing the freezing strength between pile and soil and changes the bearing capacity of pile foundation. Based on the results of high-strain dynamic test, the proportion of pile side friction resistance increased from 69.3 to 74.9% after pile foundation refreezing. The bearing capacity increased from 5237 to 7183 kN, with an increase of 37.2%.

## Data Availability

All the data pertaining to this article are included in the article.
